# Toward Fabrication of Devices Based on Graphene/Oxide Multilayers

**DOI:** 10.1021/acsaelm.3c00341

**Published:** 2023-06-06

**Authors:** Yuxuan Wang, Anais Guerenneur, Sami Ramadan, Jingle Huang, Sarah Fearn, Nomaan Nabi, Norbert Klein, Neil McN. Alford, Peter K. Petrov

**Affiliations:** † Imperial College London, London SW7 2AZ, U.K.; ‡ University College London, Gower Street, London WC1E 6BT, U.K.

**Keywords:** graphene, hexamethyldisilazane, self-assembly, deposition, sputtering

## Abstract

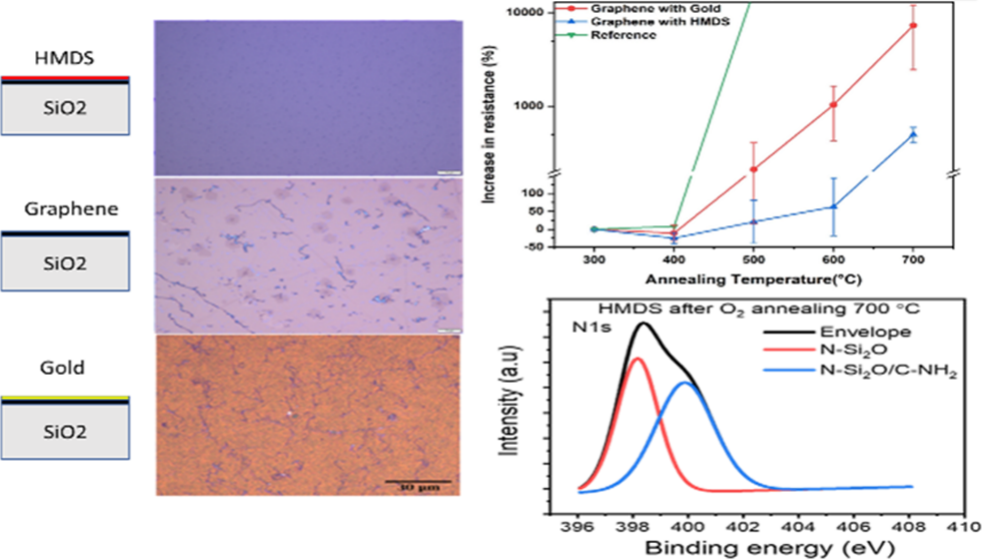

Owing to its high electrical conductivity, low density, and flexibility, graphene has great potential for use as a building block in a wide range of applications from nanoelectronics to biosensing and high-frequency devices. For many device applications, it is required to deposit dielectric materials on graphene at high temperatures and in ambient oxygen. This has been proven to be highly challenging because these conditions cause significant degradation in graphene. In this work, we investigate the degradation of graphene at elevated temperatures in an oxygen atmosphere and possible protection mechanisms to enable the growth of oxide thin films on graphene at higher temperatures. We show that coating graphene with self-assembled monolayers of hexamethyldisilazane (HMDS) prior to a high-temperature deposition can significantly reduce the damage induced. Furthermore, a graphene sample treated with HMDS displayed a weaker doping effect due to weak interaction with oxygen species than bare graphene, and a much slower rate of electrical resistance degradation was exhibited during annealing. Thus, it is a promising approach that could enable the deposition of metal oxide materials on graphene at high temperatures without significant degradation in graphene quality, which is critical for a wide range of applications.

## 1 Introduction

As a representative of the family of the wholly two-dimensional material, graphene has been used in various field applications due to its unique physical properties and superior mechanical properties.^1−8^ The most valuable properties are high electrical conductivity and high charge carrier mobility. Combined with its thickness at the angstrom scale, high electrical conductivity means that graphene can be an excellent electrode material for many different devices such as field-effect transistors,^9^ data storage devices,^10^ photodetectors,^11^ and biosensors.^12,13^

In many device-manufacturing processes such as memory and radio frequency devices, graphene is usually transferred onto the surface of a semiconducting, polymer or ceramic material, followed by the deposition or growth of the active materials on graphene. Pulsed laser deposition (PLD) and magnetron sputtering are the most used methods to grow ceramic films due to their capability to achieve high-quality films. Although low-temperature growth methods such as atomic layer deposition have been introduced for oxide layer growth on graphene,^14^ the nontoxicity, uniformity, and low cost of both PLD and magnetic sputtering still make them the most promising methods.

The growth of oxide films requires high-temperature annealing to activate the adatoms and oxygen gas to compensate for the loss of stoichiometry. However, it has been reported that the growth of an epitaxial oxide layer using PLD on top of graphene can damage graphene, which is attributed to the reactive gas atmosphere at high temperatures during deposition.^15^ Furthermore, other studies have shown that annealing graphene in oxygen can p-dope graphene even at low temperatures.^16^ As the temperature increases, it results in etch pits with nanoscale in a single graphene structure. This causes more defects in graphene and reduces its electrical conductivity.^17−19^

In this study, a thorough exploration of graphene’s behavior during high-temperature annealing in an oxygen atmosphere is conducted. The aim is to preserve the graphene structure and maintain its properties. Two methods are chosen to protect the graphene layers: metal protection and compound protection.

For metal protection, an ultrathin gold film is used due to its chemical stability and volatility in the oxygen atmosphere. Theoretically, gold should protect graphene from oxygen-related degradation. Previous studies have shown that coating graphene with a self-assembly monolayer of hexamethyldisilazane (HMDS) can also protect graphene during its exposure to plasma without degrading its electrical performance.^20^ Therefore, HMDS is chosen as the compound method during annealing.

## 2 Experimental Section

The chemical vapor deposition (CVD) graphene on copper foil was purchased from the Grolltex company. Cu foil was coated with poly (methyl methacrylate) (PMMA) to support the graphene transfer. Cu was then etched using ammonium persulfate (0.01 g/mL in H_2_O) for 12 h. The PMMA/graphene stack was then floated on two consecutive ultrapure deionized water baths to rinse the graphene surface for up to 1 h per bath. The freestanding PMMA/graphene layer was transferred to a SiO_2_/Si substrate, and PMMA/graphene on the substrate was baked on a hotplate for 2 h at 150 °C to improve the adhesion of the graphene to the substrate. PMMA was then removed using acetone for 5 h followed by isopropanol (IPA) for 5 min. The sample was then annealed at 250 °C for 5 h to remove PMMA residues on graphene. All samples were classified as sample A, sample B, or sample C. Sample A represents the pure monolayer graphene only, sample B is the monolayer graphene covered with a 2 nm gold layer using thermal evaporation, and sample C is the monolayer graphene treated with HMDS solution at room temperature for 12 h to form a uniform self-assembly monolayer on graphene surface (see the Supporting Information). To measure the electrical resistance, 16 Au electrodes with a diameter of 2 mm were formed using thermal evaporation through a shadow mask.

After that, the samples were mounted in a vacuum chamber. The chamber was pumped down to 3 × 10^–5^ Torr. Then, the sample holder was heated up to the required temperature at the rate of 30 °C/min. When the temperature reached the set point, oxygen was purged into the chamber until the partial pressure of oxygen reached 100 mTorr. The samples were then kept in this oxygen atmosphere for 10 min. After each cycle of annealing, a Raman spectrometer was used to characterize graphene. To explore the role of HMDS, X-ray photoelectron spectroscopy (XPS) and atomic force microscopy (AFM) were also used to evaluate the presence of HMDS and its influence on the graphene layer after high-temperature annealing.

Raman spectroscopy measurements were performed using a Renishaw InVia Raman spectrometer with a laser wavelength of 532 nm (excitation energy EL = *ℏwL* = 2.33 eV) which used an optical fiber, an objective lens of 100×, and NA = 0.8, resulting in a laser spot of 0.4 μm. The laser power was kept below 2 mW, and the spectral resolution was ∼3 cm^–1^; the Raman peak position was calibrated based on the Si peak position at 520.7 cm^–1^. The D, G, and 2D peaks were fitted with Lorentzian functions.

XPS experiments and measurements were performed with Kα+ and an Al radiation source (*h*ν = 1486.6 eV) in an ultrahigh vacuum chamber for spectroscopic analysis with a base pressure of 5 × 10^–8^ bar.

Secondary ion mass spectrometry (SIMS) depth profiling was carried out using an IONTOF ToF-SIMS V instrument. A 25 keV Bi^+^ ion beam was used to collect the secondary ion data in HCBM over an area of 100 μm^2^, with 128 × 128 pixels. The sample was sputtered using an Ar^+^ cluster ion beam with an energy of 10 keV and a cluster size of 1500 Ar atoms, over a sputter area of 300 μm^2^. The final crater depth was measured using a Dektak profilometer.

## 3 Results and Discussion

**Figure 1:**
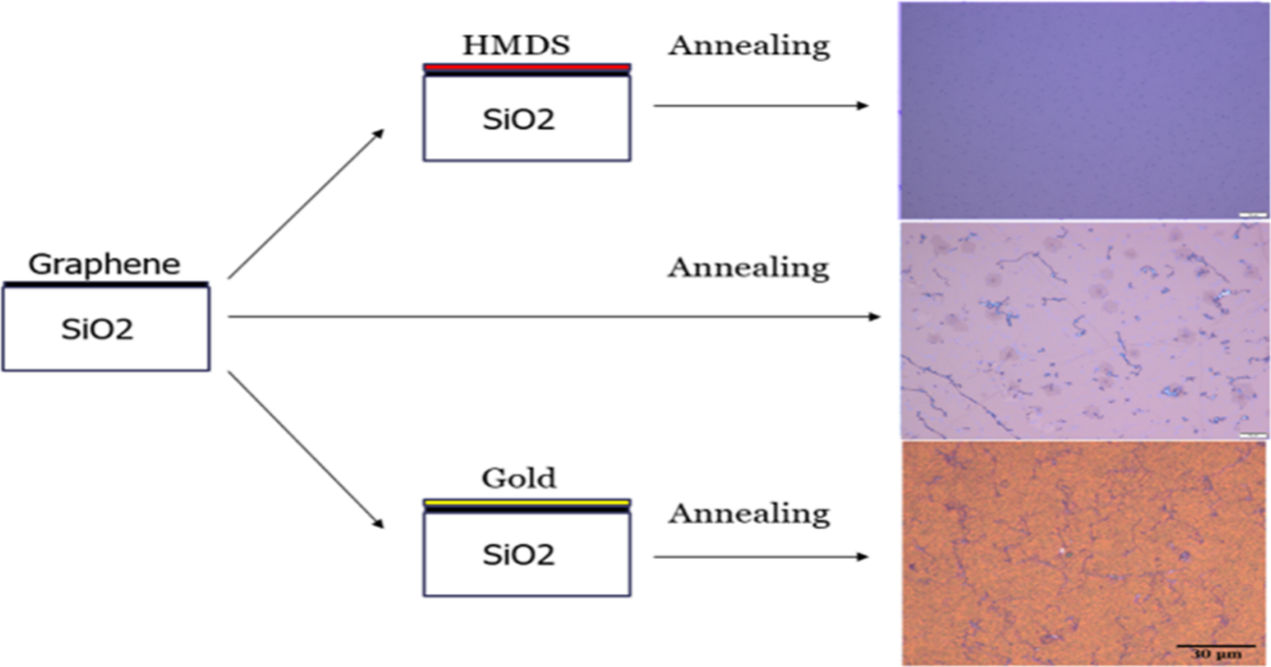
Schematics of sample structures with optical images after oxygen annealing at *T* = 700 °C. CVD graphene samples were transferred onto the silicon substrate first. Then, one of the samples was coated with HMDS, two were coated with thin gold films, and the other sample was set as a reference without any protective coating.

Figure 1 shows schematics of the sample structures and optical images after 700 °C oxygen annealing. Three types of graphene samples on Si/SiO_2_ substrates were used such as bare graphene, graphene coated with HMDS, and graphene coated with a gold layer 2 nm thick using e-beam evaporation. The properties of graphene after HMDS coating are described in detail in the Supporting Information. The samples were annealed at temperatures of 300, 400, 500, 600, and 700 °C. Electrical resistance measurements were performed after each annealing process. An increase in electrical resistance was observed for all samples after annealing above 500 °C. However, the HMDS-coated graphene exhibited the lowest degradation in electrical resistance compared to gold-coated and bare graphene. Furthermore, a decrease in electrical resistance after annealing at 400 °C was observed.

This could be attributed to the removal of PMMA residuals and improved graphene–metal contact.^21,22^ Also, in the case of the gold-coated graphene layer, one could anticipate the incorporation of gold in the graphene’s defect pits, which would effectively shorten the current percolation path.

**Figure 2:**
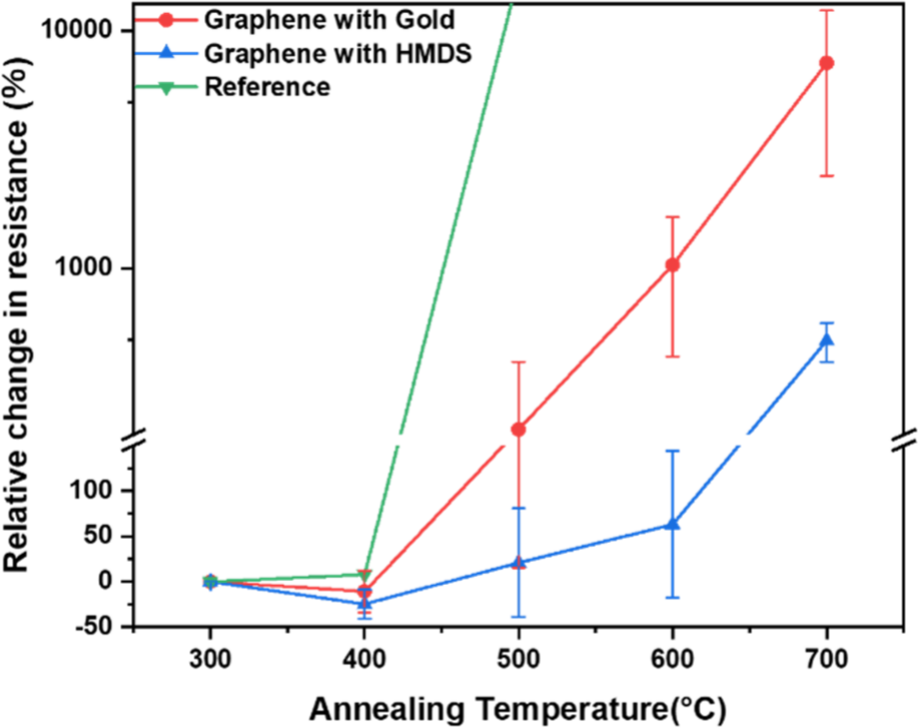
Electrical resistance measurements of the graphene samples. Graphene samples treated with HMDS show less degradation in resistance with the increase of annealing temperature compared with bare graphene and Au-coated graphene.

As the annealing temperature increased, all samples exhibited an increase in resistance which is related to the quality of graphene. The bare graphene sample showed a very high increase in resistance when the annealing temperature exceeded 400 °C, while the resistance of all the other samples shows a relatively lower increase or even a decrease at the same temperature. At higher temperatures, the resistance of the gold-coated graphene samples increased 50-fold compared to that at room temperature. In contrast, the resistance of HMDS-protected graphene only increased fivefold. The resistance result is shown in Figure 2.

**Figure 3:**
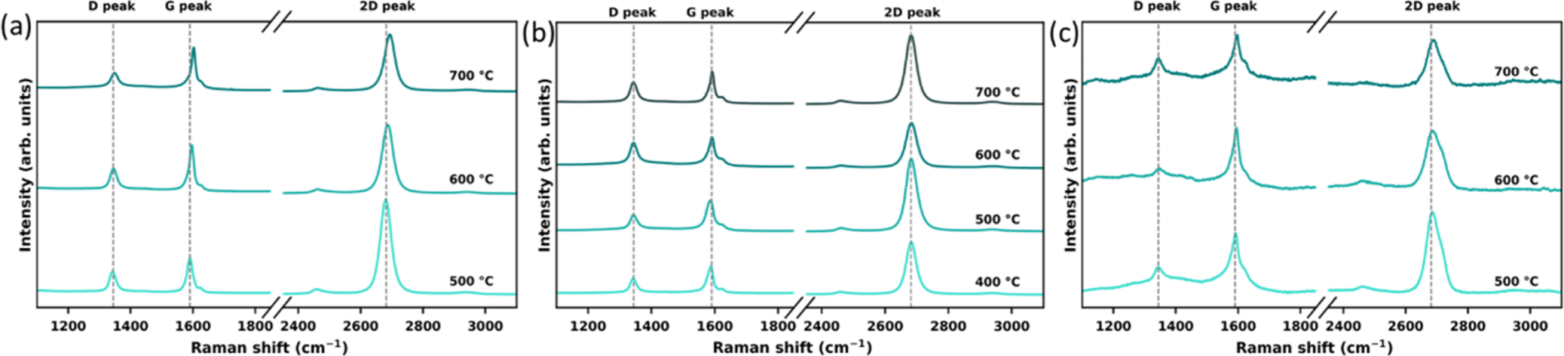
Raman spectra: (a) single-layer graphene under annealing at 500, 600, and 700 °C; (b) HMDS-treated graphene under annealing at 400, 500, 600, and 700 °C; and (c) gold-coated graphene under annealing at 500, 600, and 700 °C.

Raman spectroscopy was used to investigate the properties of graphene after annealing. Changes that occur on graphene with different protection during annealing can be expressed in terms of changes in the Raman spectrum of graphene. The Raman spectrum of as-transferred graphene shows the presence of G mode around 1594 cm^–1^ and 2D mode around 2682 cm^–1^ (Figure S2). The G mode and 2D originate from the doubly degenerate zone center phonon and second order of zone-boundary phonons.^23^ D mode was also featured around 1349 cm^–1^ which is produced by the disorder inside graphene and can be used to evaluate the defect density of graphene. After annealing, a shoulder appears in the G mode at around 1620 cm^–1^ (Figure 3). We calculated the ratio of the intensity of D mode over G mode (*I*_(D)_/*I*_(G)_) and found no evidence of significant damage to the structural properties of bare graphene and graphene coated with HMDS after annealing. The ratio of the intensity of D mode over D′ mode (*I*_(D)_/*I*_(D′)_) was also calculated according to Eckmann et al.^24^ We found that *I*_(D)_/*I*_(D′)_ in bare graphene decreases significantly with the increasing annealing temperature and the type of defects changes from sp^3^ defects to vacancy defects. In contrast, there is only a small change in *I*_(D)_/*I*_(D′)_ in graphene coated with HMDS as the annealing temperature increases. Therefore, the change of *I*_(D)_/*I*_(G)_ and *I*_(D)_/*I*_(D′)_ cannot solely explain the large increase in graphene resistance shown in Figure 2. (The experimental results supporting this statement are presented in Figure S3.)

It is worth noting that the 2D-peak mode of gold-coated graphene exhibited an asymmetric shape after 500 °C annealing. The asymmetry becomes less visible with the increase in annealing temperature. To identify the origin of this shape, we plotted the G–2D frequency modes at each annealing temperature to determine if there is any strain induced in the Au-coated graphene after annealing. After 500 °C annealing, the G–2D frequency positions are located at the strained-graphene line. Strain can reduce the symmetry in graphene and affect the orientation of graphene, resulting in the change of G and 2D modes.^25−27^ As the temperature increases, the G–2D frequency modes start to locate closer to the doping line as an indication of the reduction of strain at a higher temperature. (The results of this analysis are presented in Figure S4.)

**Figure 4:**
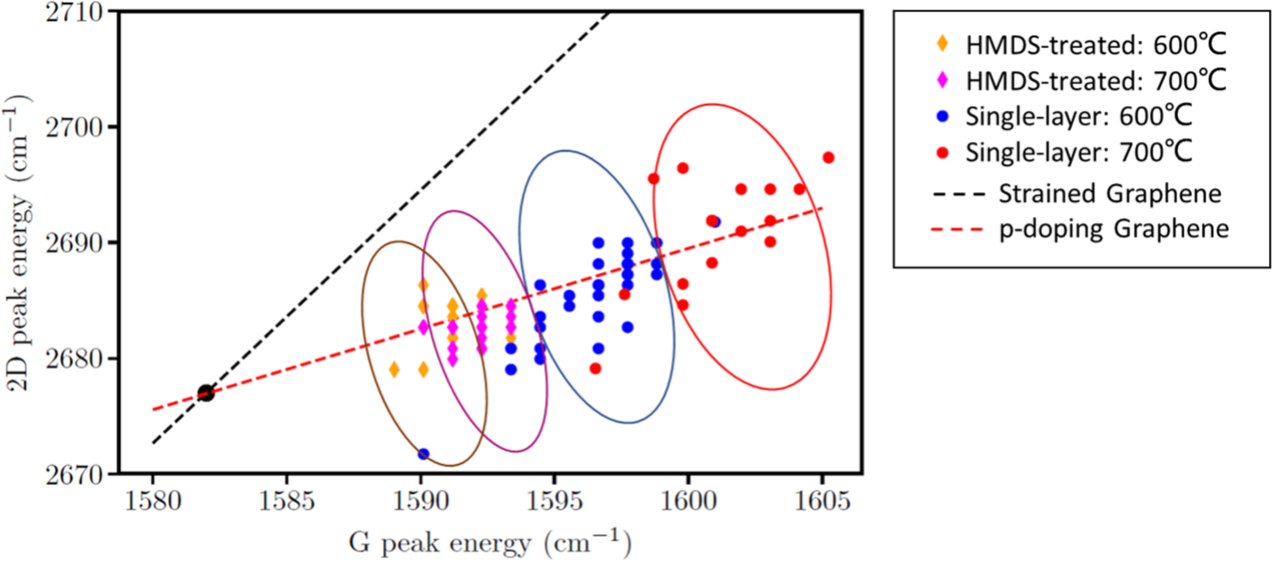
Doping—strain diagram of bare graphene and graphene with HMDS after 600 and 700 °C annealing. The red dashed line represents the doped graphene samples, while the black dashed line indicates strain in graphene. Their intersection point represents the original graphene without strain or doping.^27^ Therefore, the level of doping of the graphene sample can be described as the distance between the intersection point and the projection of the data point on the red dashed line. The data points inside the blue and red represent the G–2D peak positions for single-layer graphene at 600 and 700 °C, respectively. The data points inside the purple and orange circles represent the G–2D peak positions for HMDS-treated graphene at 600 and 700 °C, respectively.

To further investigate the impact of annealing on the doping and strain of the bare graphene and graphene treated with HMDS, the G–2D frequency mode was plotted in Figure 4. The red dashed line represents the doped graphene samples, while the black dashed line indicates strain in graphene. Their intersection point represents the original graphene without strain or doping.^27^ Therefore, the level of doping of the graphene sample can be described as the distance between the intersection point and the projection of the data point on the red dashed line. Figure 4 further confirms that graphene with HMDS exhibits lower doping levels than bare graphene. We expect that the samples are p-doped after the oxygen treatment because H_2_O and O_2_ introduce hole doping in graphene.^28^

**Figure 5:**
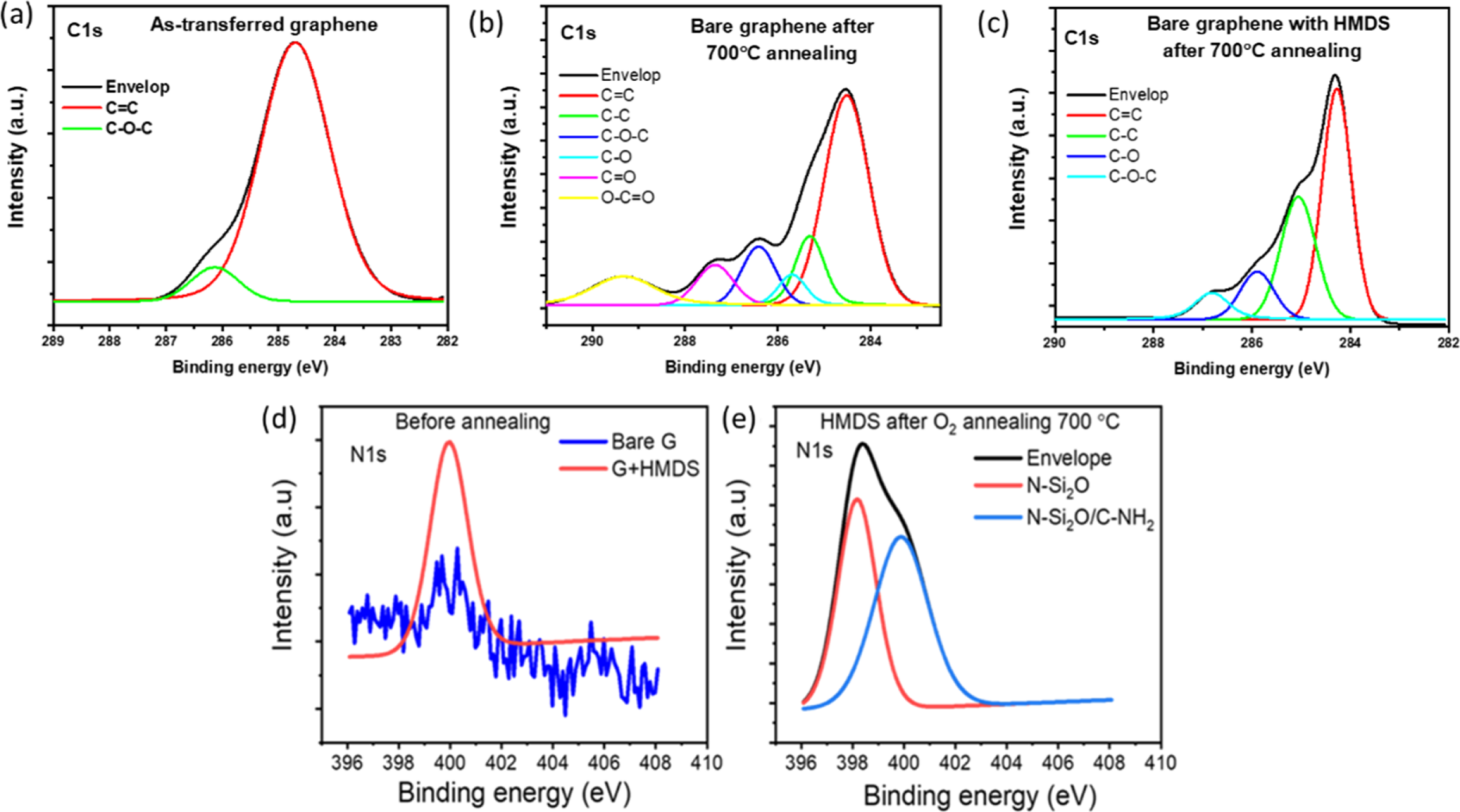
XPS results for C 1s spectra of (a) as-transferred graphene at room temperature and (b) bare graphene after 700 °C annealing. (c) Bare graphene with HMDS after 700 °C annealing. N 1s spectra of (d) graphene with HMDS before annealing and (e) after annealing. A C–O–C peak with increased density was presented in bare graphene after annealing compared with the graphene before annealing. The O–C=O bond is observed in the C 1s spectrum of bare graphene after annealing The N 1s signal from graphene with HMDS before annealing indicates the existence of HMDS. After annealing, most of the HMDS has been removed, but some of its residuals are still present, confirming that the HMDS can protect graphene.

The low resistance of as-transferred graphene coated with HMDS suggests that the formation of the HMDS layer by self-assembly possibly still exists even after high-temperature annealing. This layer acts as a barrier that may prevent oxygen diffusion to the graphene film. To further explore this assumption, we performed XPS measurements to assess the elemental composition of graphene at room temperature and after annealing at 700 °C. After annealing at 700 °C, the XPS C 1s spectrum reveals the presence of a C–O–C peak with increased density compared with graphene before annealing. Another O–C=O bond is observed in the C 1s spectrum of bare graphene after annealing (Figure 5a,b). Interestingly, after annealing, the O–C=O peak was not observed in graphene treated with the HMDS sample (Figure 5c). Furthermore, high-temperature annealing above 500 °C can increase the coupling between SiO_2_ and graphene and hence increase the level of p-doping.^29^ Therefore, the formation of O–C=O and C–O–C bonds and the increased coupling between the substrate and graphene at high temperatures could contribute to the increase of scattering in graphene after annealing and possibly could play a role in the increase of the resistance.

Finally, the N 1s peak of graphene with HMDS after annealing shows the presence of N–Si_2_O and C–NH_2_ (Figure 5d,e), which are compounds related to HMDS. These residuals indicate that the HMDS layer still provides protection even after 700 °C annealing.

**Figure 6:**
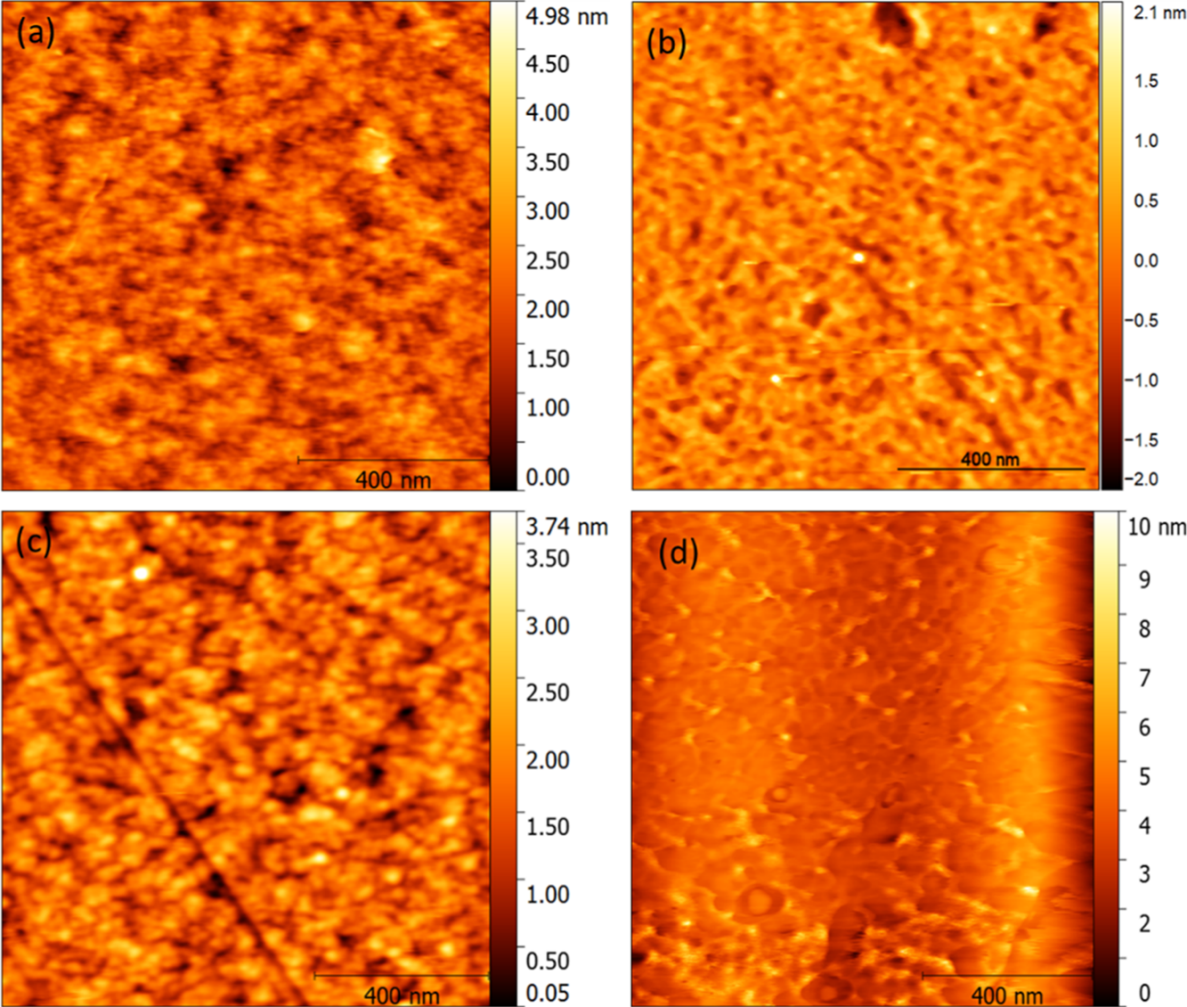
AFM images of bare graphene and graphene treated with HMDS before and after annealing: (a) bare graphene before annealing. (b) Graphene with HMDS before annealing. (c) Bare graphene after annealing at 450 °C and (d) graphene with HMDS after annealing at 450 °C. The bare graphene exhibits more defects after annealing at 450 °C, while the graphene with HMDS has fewer defects.

To confirm this assumption, AFM was conducted. The AFM profile shows that an additional film exists on the surface of the annealed graphene sample with HMDS (Figure 6c), and this film looks like the remnants of HMDS after high-temperature annealing. The values of surface roughness of the bare graphene and graphene with HMDS were 0.9 and 2.3 nm, respectively. Also, bare graphene has a higher density of void structures compared with graphene treated with HMDS. Thus, the AFM studies confirm that HMDS provides good protection for graphene during annealing.

To fully integrate HMDS during device manufacture, the HMDS layer should not affect the quality of the graphene/thin film stack. We expect that, during physical vapor deposition such as in magnetron sputtering and PLD, species with high energy may remove the HMDS on the self-assembled monolayers (SAMs) during the thin-film growth process.

**Figure 7:**
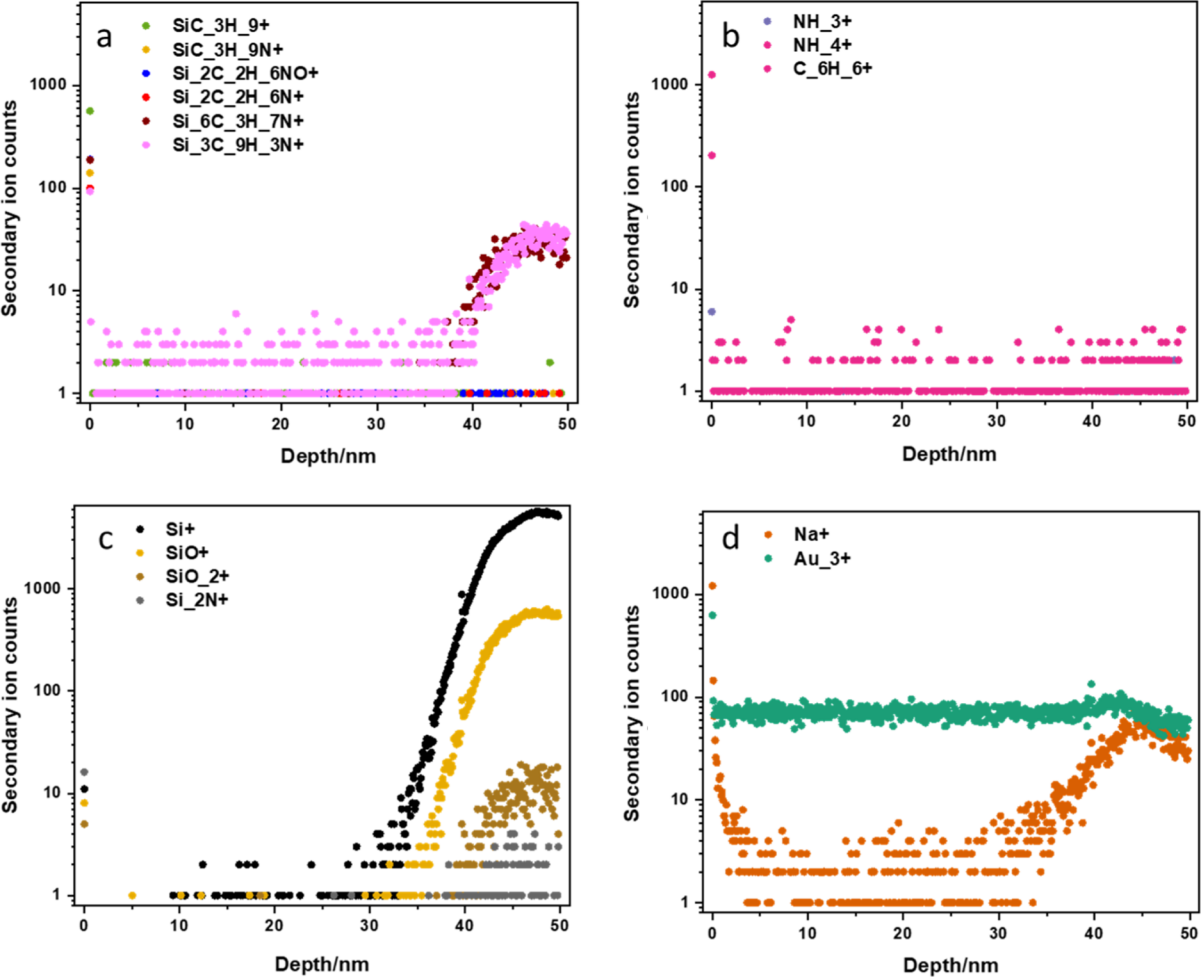
SIMS results for Au/HMDS/graphene: (a) for potential silicon-organic compounds, with the highest counts failing even to reach 100 indicating their scarcity; (b) low levels for other possible common chemicals suggesting no such contamination in the sample; and (c,d) strong signals for silicon, silica, and graphene confirm the known structure of graphene on the Si/SiO_2_ substrate.

To investigate this hypothesis, a thin gold film was grown on HMDS-covered graphene using magnetron sputtering and SIMS was then employed to investigate the sample’s elemental depth profile. The results obtained are shown in Figure 7.

The SIMS results show that the signal for gold remains at a constant level throughout the entire film thickness of about 50 nm. The signal for Si/SiO_2_ is enhanced across the thickness of the sample which approaches the silicon substrate underneath the graphene layer. The signal for Na^+^ which represents the presence of graphene shows a slight increase at a depth of 45–50 nm, which is consistent with the existence of a single layer of graphene underneath the gold film. Some organic compounds that contain Si were also detected; however, these signals are likely to be related to PMMA with residues from the transfer process in the commercial CVD graphene to silicon substrates. Thus, it is concluded that the characterization using SIMS detects hardly any signal that indicates the presence of HMDS after the magnetron sputtering.

## 4 Conclusions

This study demonstrates that graphene’s electrical resistance and integrity can be preserved during a treatment typical for oxide thin-film deposition, i.e., high temperatures in an oxygen atmosphere. A series of electrical resistance measurements and surface analyses using XPS and AFM show that the SAMs of HMDS can provide better protection to graphene compared with a thin layer of gold. Furthermore, the SIMS results show strong evidence that HMDS disappears during a consecutive magnetron sputtering process. Thus, HMDS can provide adequate protection of graphene during an oxide-thin-film deposition process without affecting the properties of the manufactured device.

## Supporting Information Available

AFM image of HMDS graphene and more analysis about Raman spectra (PDF)
