# Supplementary material for: Toward Fabrication of Devices Based on Graphene/Oxide Multilayers
Source: ACS Appl Electron Mater. 2023 Jun 6;5(6):3261–7. doi: 10.1021/acsaelm.3c00341 (PMC10308813; doi:10.1021/acsaelm.3c00341)
Supplement: Supplementary file 1 — el3c00341_si_001.pdf [file el3c00341_si_001.pdf]

Supporting information

Towards fabrication of devices based on graphene/oxide multilayers

*Yuxuan Wang[a], Anais Guerenneur[a#], Sami Ramadan[a], Jingle Huang[b], Sarah Fearn[a], Nomaan Nabi[a], Norbert Klein[a], Neil McN. Alford [a] and Peter K. Petrov[a]**

[a] Imperial College London, London SW7 2AZ, UK

[b] University College London, Gower St, London WC1E 6BT, UK

*Email: p.petrov@imperial.ac.uk

Supplementary Results

S 1 HMDS treatment of graphene surface

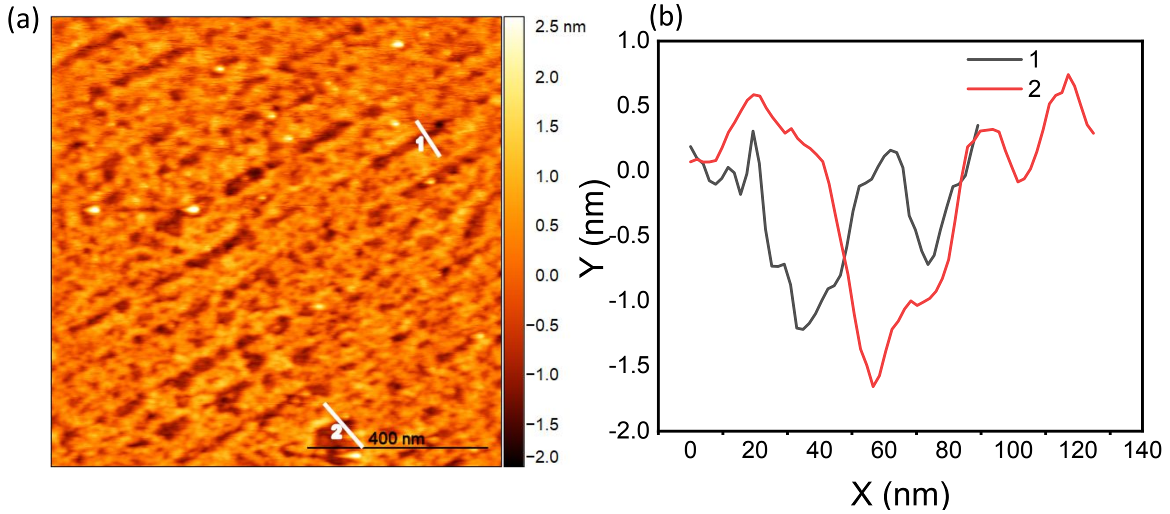

Figure S1.(a) AFM characteristics of Gr after HMDS treatment. (b) Line profiles show the thickness of HMDS on Gr. The average thickness of HMDS on Gr is 1.5 nm.

S 2 Raman spectra of as-transferred graphene and as-transferred graphene with HMDS and distribution of G and 2D mode

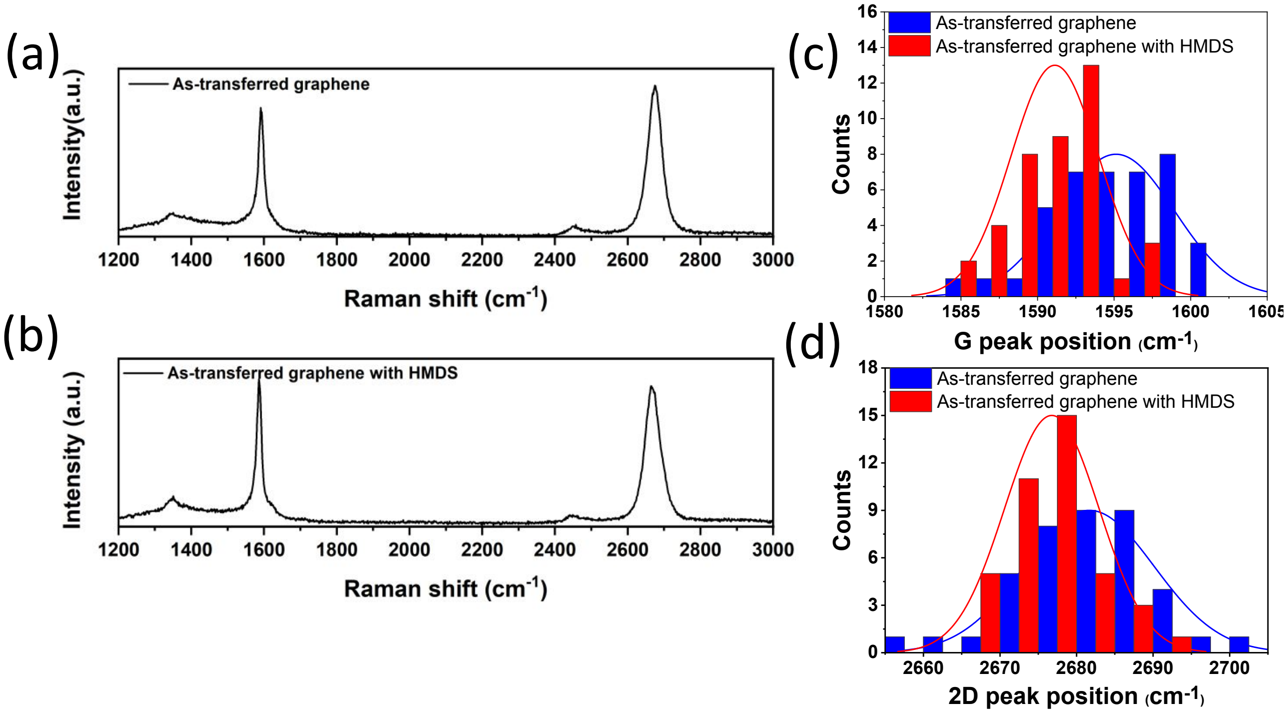

Figure S2.(a) Raman spectra of as-transferred graphene. (b) Raman spectra of as-transferred graphene with HMDS treatment. (c) The distribution of the G peak position of as-transferred and HMDS-treated graphene. (b) The distribution of the 2D position of as-transferred and HMDS-treated graphene. The G and 2D peaks are blue-shifted after HMDS treatment as an indication of reduction of p-doping due to the removal of water molecules from the surface of graphene after HMDS treatment.

S 3 Analysis of defect peaks intensities ratio in graphene and graphene coated with HMDS after annealing

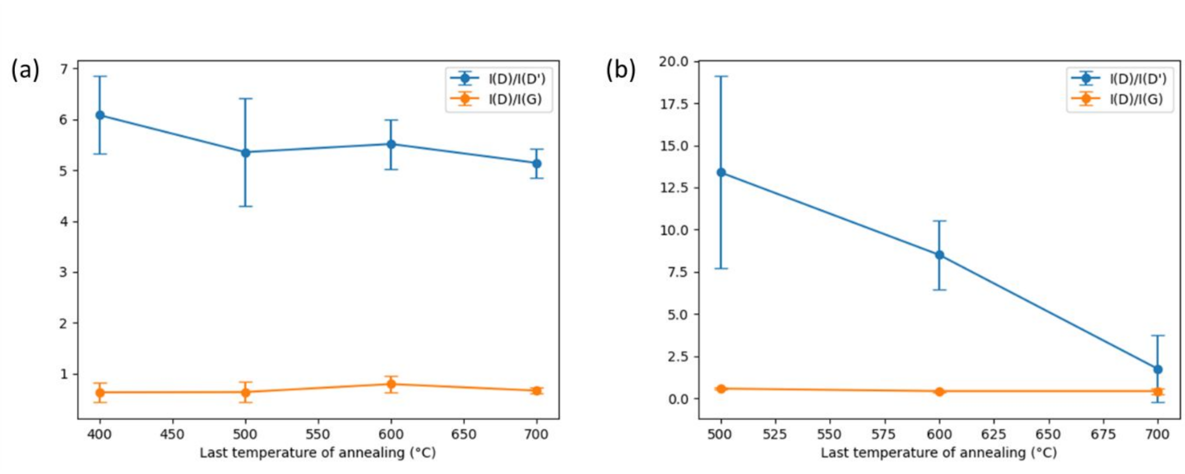

Figure S3. (a). The ratio of intensity of D mode over D' mode ($I_{(D)}/I_{(D')}$) and D mode over G mode ($I_{(D)}/I_{(G)}$) in as-transferred graphene with HMDS. (b). The ratio of the intensity of D mode over D' mode and D mode over G mode in as-transferred graphene. The $I_{(D)}/I_{(G)}$ barely shows any significant change in both as-transferred graphene and graphene with HMDS. However, $I_{(D)}/I_{(D')}$ in as-transferred graphene reveals the change of type of defects from sp^3 defects to vacancy defects as the annealing temperature increase. The same index in as-transferred graphene with HMDS shows a slight decrease with the increasing annealing temperature which suggests the protective capability of HMDS on pristine graphene from the annealing.

S 4 Analysis of Au-coated graphene

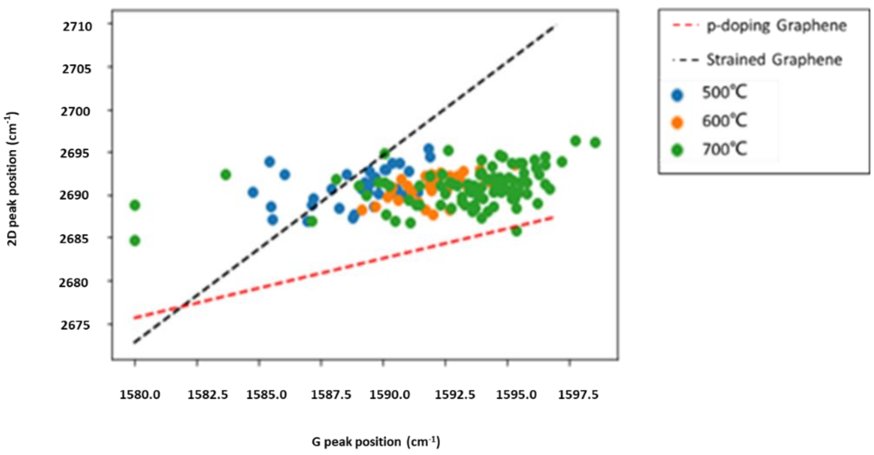

Figure S4. The impact of annealing on the frequency modes of G and 2D modes for Au-coated graphene. The Black dashed line represents the G-2D frequency position of carrier-free graphene, and the red dashed line represents the G-2D frequency position of strain-free graphene. The blue, orange and green dots represent the G-2D frequency positions for the samples annealed at 500˚C, 600˚C and 700˚C, respectively. As the annealing temperature increase, the doping level of the graphene sample also increases, and strain is reduced. This is indicated by the longer projection distance between data points and intersection points along the red dashed line.
